# *N*-(3-Meth­oxy­phen­yl)-4-{4-methyl-2-[(meth­yl)(4-methyl­phen­yl)amino]-1,3-thia­zol-5-yl}pyrimidin-2-amine

**DOI:** 10.1107/S1600536810053559

**Published:** 2011-01-08

**Authors:** Hai-Bo Shi, Hai-Bo Li, Wei-Xiao Hu

**Affiliations:** a Zhejiang Pharmaceutical College, Ningbo 315100, People’s Republic of China; b College of Pharmaceutical Science, Zhejiang University of Technology, Hangzhou 310032, People’s Republic of China; c Nantong Center for Disease Control and Prevention, Nantong 226007, People’s Republic of China

## Abstract

The asymmetric unit of the title compound, C_23_H_23_N_5_OS, contains two independent mol­ecules. In one mol­ecule, the thia­zole and pyrimidine rings are almost co-planar, making a dihedral angle of 2.48 (8)°. In the other mol­ecule, the corresponding dihedral angle is 12.82 (8)°. The crystal structure is stabilized by weak inter­molecular N—H⋯N and C—H⋯O inter­actions that extend along the *b* axis.

## Related literature

For general background to the biological activity of thia­zole derivatives, see: Narayana *et al.* (2004 ▶). For the synthesis of the title compound, see: Bredereck *et al.* (1964 ▶). 
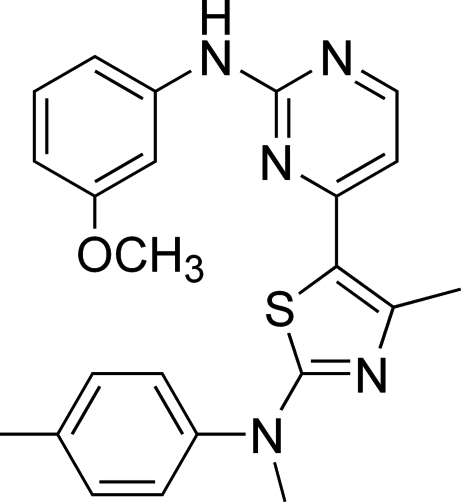


## Experimental

### 

#### Crystal data

C_23_H_23_N_5_OS
*M* *_r_* = 417.52
Triclinic, 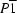

*a* = 10.280 (3) Å
*b* = 12.413 (2) Å
*c* = 17.671 (4) Å
α = 74.290 (9)°
β = 87.96 (1)°
γ = 68.281 (7)°
*V* = 2011.3 (8) Å^3^
*Z* = 4
Mo *K*α radiation
μ = 0.19 mm^−1^
*T* = 153 K
0.40 × 0.40 × 0.23 mm

#### Data collection

Rigaku AFC10/Saturn724+ diffractometer
Absorption correction: multi-scan (*CrystalClear*; Rigaku/MSC, 2008 ▶) *T*_min_ = 0.929, *T*_max_ = 0.958
19475 measured reflections
9061 independent reflections
6741 reflections with *I* > 2σ(*I*)
*R*_int_ = 0.028

#### Refinement

*R*[*F* ^2^ > 2σ(*F* ^2^)] = 0.043
*wR*(*F* ^2^) = 0.115
*S* = 1.03
9061 reflections
551 parameters
H-atom parameters constrained
Δρ_max_ = 0.34 e Å^−3^
Δρ_min_ = −0.25 e Å^−3^

### 

Data collection: *CrystalClear* (Rigaku/MSC, 2008 ▶); cell refinement: *CrystalClear*; data reduction: *CrystalClear*; program(s) used to solve structure: *SHELXS97* (Sheldrick, 2008 ▶); program(s) used to refine structure: *SHELXL97* (Sheldrick, 2008 ▶); molecular graphics: *SHELXTL* (Sheldrick, 2008 ▶); software used to prepare material for publication: *publCIF* (Westrip, 2010 ▶).

## Supplementary Material

Crystal structure: contains datablocks I, global. DOI: 10.1107/S1600536810053559/ng5080sup1.cif

Structure factors: contains datablocks I. DOI: 10.1107/S1600536810053559/ng5080Isup2.hkl

Additional supplementary materials:  crystallographic information; 3D view; checkCIF report

## Figures and Tables

**Table 1:** Hydrogen-bond geometry (Å, °)

*D*—H⋯*A*	*D*—H	H⋯*A*	*D*⋯*A*	*D*—H⋯*A*
N5—H0⋯N3′^i^	0.88	2.27	3.151 (2)	177
N5′—H0′⋯N3^i^	0.88	2.24	3.089 (2)	164
C22—H22*B*⋯O1^ii^	0.98	2.57	3.500 (2)	159

Symmetry codes: (i) 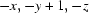; (ii) 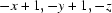.

**Fig. 1.**
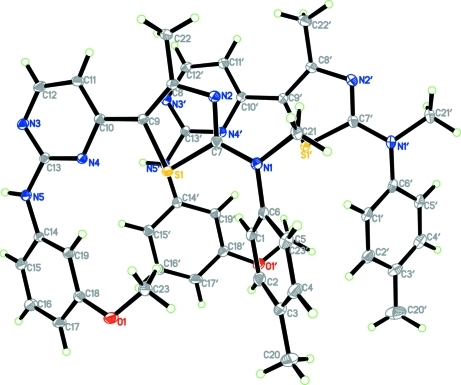
The structure of (I), shown with 30% probability displacement ellipsoids.

**Fig. 2.**
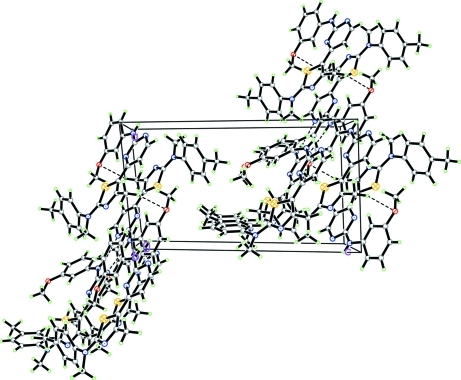
Packing of the molecules down b axis. Dashed lines denote intermolecular N—H···N and C—H···O hydrogen bonds.

## supplementary crystallographic information

### Comment

Thiazole derivatives are found to be associated with various biological activities (Narayana *et al.*, 2004). In order to further study the structure-activity relationship (SAR) of the thiazolyl-pyrimidine derivatives, we introduced arylamino group into 2-position of thiazole ring of thiazolyl-pyrimidine according to the general pyrimidine condensation method of Bredereck (Bredereck *et al.*, 1964). But, it was found that the obtained compound was not desired compound that confirmed by ^1^H NMR, MS. So, the structure of (I) was further determined using single-crystal X-ray diffraction.

The molecular structure of (I) is illustrated in Fig. 1. The benzene ring makes opposite angles with thiazole-pyrimidine ring. The thiazole ring (S1/C7/N2/C8/C9) and the pyrimidine ring (C10/C11/C12/N3/C13/N4) are almost planar, with a dihedral angle of 2.48 (8)°. The aniline rings (C1/C2/C3/C4/C5/C6/N1) and (C14/C15/C16/C17/C18/C19/N5) make dihedral angles of 53.06 (8) Å and 19.21 (8) Å with the thiazole ring, respectively. In contrast, in the other independent molecule, the thiazole ring (S1'/C7'/N2'/C8'/C9') and the pyrimidine ring (C10'/C11'/C12'/N3'/C13'/N4') make a dihedral angle of 12.82 (8)°. The aniline rings (C1'/C2'/C3'/C4'/C5'/C6'/N1') and (C14'/C15'/C16'/C17'/C18'/C19'/N5') make dihedral angles of 51.09 (9) Å and 29.07 (9) Å with the thiazole ring, respectively. Moreover, there exist different bond lengths in the two independent molecules due to different intermolecular hydrogen bonding interactions (Table 1). The crystal structure is stabilized by intermolecular weak N—H···N and C—H···O interactions, which extending down b axis.

### Experimental

A mixture of

3-Dimethylamino-1-[2-(methyl-*p*-tolyl-amino)-4-methyl-thiazol-5-yl]-propenone (1.575 g, 5 mmol) and NaOH (0.2 g, 5 mmol) in 2-methoxylethanol (35 ml) was treated with *N*-(3-Methoxy-phenyl)-guanidine carbonate (1.476 g, 6.5 mmol). The reaction mixture was heated at 383 K under N_2_ for 11 h. After concentration, the residue was filtered and washed liberally with ethanol and water. Recrystallization from THF affored the title compound as brown-yellow crystals, 0.97 g, m.p.453–456 K, yield 46.7%.Since the crystal product was not found to be suitable for X-ray diffraction studies,a few crystals were dissolved in 2-butanone, which was allowed to evaporate slowly to give yellow crystals of (I) suitable for X-ray diffraction studies. ^1^H NMR(CDCl_3_, TMS, 400 MHz, δp.p.m.): 8.27 (s, 1H, *J* = 4.8 Hz, py—H), 7.53 (s, 1H, Ar—H), 7.29–7.07 (m, 5H, Ar—H), 6.87 (d, 1H,*J* = 8.0 Hz, Ar—H), 6.83 (d, 1H, *J* = 5.2 Hz, py—H), 6.54 (d, 1H, *J* = 8.0 Hz, Ar—H), 3.64 (s, 3H, OCH_3_), 3.54 (s, 3H, CH_3_), 2.60 (s, 3H, CH_3_), 2.41 (s, 3H, CH_3_). EIMS m/*z* (%): 417 (*M*^+^,100), 402 (7), 311 (12), 105 (9), 91 (10), 73 (12), 57 (17).

### Refinement

All H atoms were placed in calculated positions (C—H 0.95–0.98 Å, N—H 0.87–0.89 Å) and refined as riding with *U*_iso_(H) = 1.2–1.22Ueq of the parent atom.

### Crystal data

**Table:**

C_23_H_23_N_5_OS	*Z* = 4
*M**_r_* = 417.52	*F*(000) = 880
Triclinic, *P*1	*D*_x_ = 1.379 Mg m^−^^3^
Hall symbol: -P 1	Melting point = 456–453 K
*a* = 10.280 (3) Å	Mo *K*α radiation, λ = 0.71073 Å
*b* = 12.413 (2) Å	Cell parameters from 5913 reflections
*c* = 17.671 (4) Å	θ = 3.0–27.5°
α = 74.290 (9)°	µ = 0.19 mm^−^^1^
β = 87.96 (1)°	*T* = 153 K
γ = 68.281 (7)°	Block, yellow
*V* = 2011.3 (8) Å^3^	0.40 × 0.40 × 0.23 mm

### Data collection

**Table:**

Rigaku AFC10/Saturn724+ diffractometer	9061 independent reflections
Radiation source: fine-focus sealed tube	6741 reflections with *I* > 2σ(*I*)
graphite	*R*_int_ = 0.028
Detector resolution: 28.5714 pixels mm^-1^	θ_max_ = 27.5°, θ_min_ = 3.1°
phi and ω scans	*h* = −13→13
Absorption correction: multi-scan (*CrystalClear*; Rigaku/MSC, 2008)	*k* = −16→12
*T*_min_ = 0.929, *T*_max_ = 0.958	*l* = −22→21
19475 measured reflections	

### Refinement

**Table:**

Refinement on *F*^2^	Primary atom site location: structure-invariant direct methods
Least-squares matrix: full	Secondary atom site location: difference Fourier map
*R*[*F*^2^ > 2σ(*F*^2^)] = 0.043	Hydrogen site location: inferred from neighbouring sites
*wR*(*F*^2^) = 0.115	H-atom parameters constrained
*S* = 1.03	*w* = 1/[σ^2^(*F*_o_^2^) + (0.0641*P*)^2^] where *P* = (*F*_o_^2^ + 2*F*_c_^2^)/3
9061 reflections	(Δ/σ)_max_ = 0.002
551 parameters	Δρ_max_ = 0.34 e Å^−^^3^
0 restraints	Δρ_min_ = −0.25 e Å^−^^3^

### Special details

**Table:**

Geometry. All e.s.d.'s (except the e.s.d. in the dihedral angle between two l.s. planes) are estimated using the full covariance matrix. The cell e.s.d.'s are taken into account individually in the estimation of e.s.d.'s in distances, angles and torsion angles; correlations between e.s.d.'s in cell parameters are only used when they are defined by crystal symmetry. An approximate (isotropic) treatment of cell e.s.d.'s is used for estimating e.s.d.'s involving l.s. planes.
Refinement. Refinement of *F*^2^ against ALL reflections. The weighted *R*-factor *wR* and goodness of fit *S* are based on *F*^2^, conventional *R*-factors *R* are based on *F*, with *F* set to zero for negative *F*^2^. The threshold expression of *F*^2^ > σ(*F*^2^) is used only for calculating *R*-factors(gt) *etc*. and is not relevant to the choice of reflections for refinement. *R*-factors based on *F*^2^ are statistically about twice as large as those based on *F*, and *R*- factors based on ALL data will be even larger.

### Fractional atomic coordinates and isotropic or equivalent isotropic displacement parameters (Å^2^)

**Table:**

	*x*	*y*	*z*	*U*_iso_*/*U*_eq_	
S1	0.50315 (4)	0.38212 (4)	0.11927 (2)	0.02004 (11)	
O1	0.31361 (12)	0.76460 (10)	0.16457 (7)	0.0297 (3)	
N1	0.74892 (14)	0.22921 (12)	0.20606 (8)	0.0237 (3)	
N2	0.66177 (14)	0.16781 (12)	0.11226 (8)	0.0215 (3)	
N3	0.07766 (14)	0.54098 (12)	−0.08838 (8)	0.0226 (3)	
N4	0.25101 (14)	0.50344 (11)	0.01314 (7)	0.0183 (3)	
C1	0.61409 (18)	0.34713 (15)	0.29291 (9)	0.0233 (4)	
H1	0.5369	0.3255	0.2855	0.028*	
C2	0.60597 (19)	0.41897 (16)	0.34179 (10)	0.0287 (4)	
H2	0.5222	0.4465	0.3674	0.034*	
C3	0.7163 (2)	0.45221 (16)	0.35466 (10)	0.0301 (4)	
C4	0.8380 (2)	0.40956 (16)	0.31677 (10)	0.0307 (4)	
H4	0.9158	0.4298	0.3252	0.037*	
C5	0.84791 (18)	0.33830 (16)	0.26710 (10)	0.0264 (4)	
H5	0.9317	0.3108	0.2414	0.032*	
C6	0.73559 (17)	0.30653 (14)	0.25448 (9)	0.0202 (3)	
C7	0.64954 (17)	0.24899 (14)	0.14957 (9)	0.0204 (3)	
C8	0.55242 (17)	0.20873 (14)	0.05698 (9)	0.0203 (3)	
C9	0.45492 (16)	0.32321 (14)	0.05084 (9)	0.0186 (3)	
C10	0.32638 (16)	0.39460 (14)	0.00112 (9)	0.0186 (3)	
C11	0.27980 (18)	0.35759 (15)	−0.05700 (10)	0.0257 (4)	
H11	0.3320	0.2823	−0.0668	0.031*	
C12	0.15554 (18)	0.43451 (15)	−0.09934 (10)	0.0266 (4)	
H12	0.1230	0.4102	−0.1391	0.032*	
C13	0.13195 (16)	0.57067 (14)	−0.03208 (9)	0.0191 (3)	
C14	0.06599 (17)	0.74316 (14)	0.02929 (9)	0.0202 (3)	
C15	−0.05486 (18)	0.83747 (15)	0.03962 (10)	0.0272 (4)	
H15	−0.1416	0.8547	0.0126	0.033*	
C16	−0.04857 (19)	0.90526 (16)	0.08867 (10)	0.0316 (4)	
H16	−0.1305	0.9703	0.0941	0.038*	
C17	0.07593 (19)	0.87930 (15)	0.12995 (10)	0.0278 (4)	
H17	0.0801	0.9256	0.1641	0.033*	
C18	0.19478 (17)	0.78470 (14)	0.12089 (10)	0.0225 (4)	
C19	0.19246 (17)	0.71745 (14)	0.07009 (9)	0.0213 (4)	
H19	0.2757	0.6549	0.0632	0.026*	
C20	0.7059 (3)	0.52907 (19)	0.40976 (12)	0.0483 (6)	
H20A	0.7917	0.5462	0.4096	0.058*	
H20B	0.6946	0.4857	0.4633	0.058*	
H20C	0.6248	0.6051	0.3921	0.058*	
C21	0.87705 (18)	0.12023 (16)	0.21626 (11)	0.0303 (4)	
H21A	0.8514	0.0509	0.2184	0.036*	
H21B	0.9303	0.1054	0.2654	0.036*	
H21C	0.9348	0.1318	0.1717	0.036*	
C22	0.55248 (19)	0.12289 (15)	0.01110 (10)	0.0276 (4)	
H22A	0.6287	0.0447	0.0330	0.033*	
H22B	0.5664	0.1557	−0.0442	0.033*	
H22C	0.4624	0.1123	0.0147	0.033*	
C23	0.42224 (18)	0.64788 (16)	0.17922 (11)	0.0312 (4)	
H23A	0.3823	0.5860	0.1995	0.037*	
H23B	0.4933	0.6396	0.2182	0.037*	
H23C	0.4658	0.6378	0.1301	0.037*	
S1'	0.62905 (4)	0.02186 (4)	0.35481 (2)	0.02269 (11)	
O1'	0.32556 (14)	0.34116 (11)	0.45343 (7)	0.0364 (3)	
N1'	0.86877 (15)	−0.12792 (13)	0.44911 (8)	0.0268 (3)	
N2'	0.81090 (15)	−0.18076 (13)	0.34154 (9)	0.0273 (3)	
N3'	0.21894 (15)	0.16941 (12)	0.14055 (8)	0.0236 (3)	
N4'	0.39489 (14)	0.14313 (11)	0.23753 (8)	0.0188 (3)	
C1'	0.80415 (18)	0.07463 (16)	0.46503 (10)	0.0269 (4)	
H1'	0.8030	0.1065	0.4096	0.032*	
C2'	0.7700 (2)	0.15134 (17)	0.51301 (11)	0.0312 (4)	
H2'	0.7424	0.2357	0.4895	0.037*	
C3'	0.7748 (2)	0.10854 (18)	0.59446 (11)	0.0336 (4)	
C4'	0.8157 (2)	−0.01508 (18)	0.62604 (11)	0.0324 (4)	
H4'	0.8218	−0.0473	0.6816	0.039*	
C5'	0.84775 (19)	−0.09272 (17)	0.57929 (10)	0.0279 (4)	
H5'	0.8753	−0.1770	0.6030	0.033*	
C6'	0.84012 (17)	−0.04864 (16)	0.49739 (10)	0.0250 (4)	
C7'	0.78435 (17)	−0.10428 (15)	0.38390 (10)	0.0237 (4)	
C8'	0.70529 (18)	−0.14324 (15)	0.28392 (10)	0.0259 (4)	
C9'	0.59585 (17)	−0.03712 (14)	0.28235 (9)	0.0212 (4)	
C10'	0.46677 (17)	0.02952 (14)	0.23224 (9)	0.0204 (3)	
C11'	0.41421 (19)	−0.01837 (16)	0.18333 (10)	0.0269 (4)	
H11'	0.4618	−0.0989	0.1807	0.032*	
C12'	0.29073 (19)	0.05571 (15)	0.13900 (10)	0.0270 (4)	
H12'	0.2542	0.0242	0.1052	0.032*	
C13'	0.27571 (17)	0.20653 (14)	0.19190 (9)	0.0195 (3)	
C14'	0.20829 (16)	0.38181 (14)	0.24965 (9)	0.0194 (3)	
C15'	0.13941 (18)	0.50655 (15)	0.22759 (10)	0.0260 (4)	
H15'	0.0979	0.5474	0.1755	0.031*	
C16'	0.1311 (2)	0.57126 (16)	0.28114 (12)	0.0328 (4)	
H16'	0.0825	0.6563	0.2658	0.039*	
C17'	0.1926 (2)	0.51390 (16)	0.35649 (11)	0.0327 (4)	
H17'	0.1850	0.5586	0.3935	0.039*	
C18'	0.26553 (18)	0.39037 (16)	0.37766 (10)	0.0251 (4)	
C19'	0.27451 (17)	0.32300 (15)	0.32526 (9)	0.0210 (3)	
H19'	0.3249	0.2382	0.3405	0.025*	
C20'	0.7379 (3)	0.1922 (2)	0.64597 (13)	0.0567 (7)	
H20D	0.6760	0.1704	0.6856	0.068*	
H20E	0.6897	0.2754	0.6136	0.068*	
H20F	0.8238	0.1856	0.6724	0.068*	
C21'	0.96868 (19)	−0.25356 (16)	0.47869 (12)	0.0358 (5)	
H21D	0.9171	−0.3061	0.5006	0.043*	
H21E	1.0339	−0.2588	0.5199	0.043*	
H21F	1.0214	−0.2794	0.4353	0.043*	
C22'	0.7241 (2)	−0.22037 (18)	0.22881 (12)	0.0422 (5)	
H22D	0.8231	−0.2735	0.2320	0.051*	
H22E	0.6952	−0.1686	0.1747	0.051*	
H22F	0.6664	−0.2693	0.2438	0.051*	
C23'	0.4145 (2)	0.21765 (16)	0.47661 (10)	0.0311 (4)	
H23D	0.3615	0.1684	0.4712	0.037*	
H23E	0.4501	0.1951	0.5317	0.037*	
H23F	0.4936	0.2039	0.4430	0.037*	
N5	0.05080 (14)	0.68186 (12)	−0.02343 (8)	0.0225 (3)	
H0	−0.0238	0.7215	−0.0569	0.036 (6)*	
N5'	0.19792 (14)	0.32157 (12)	0.19486 (8)	0.0212 (3)	
H0'	0.1314	0.3641	0.1567	0.029 (5)*	

### Atomic displacement parameters (Å^2^)

**Table:**

	*U*^11^	*U*^22^	*U*^33^	*U*^12^	*U*^13^	*U*^23^
S1	0.01671 (19)	0.0193 (2)	0.0227 (2)	−0.00196 (15)	−0.00337 (16)	−0.00978 (17)
O1	0.0243 (6)	0.0262 (7)	0.0397 (7)	−0.0040 (5)	−0.0083 (6)	−0.0170 (6)
N1	0.0176 (7)	0.0247 (8)	0.0249 (7)	0.0006 (6)	−0.0038 (6)	−0.0121 (6)
N2	0.0193 (7)	0.0222 (7)	0.0209 (7)	−0.0032 (5)	−0.0003 (6)	−0.0090 (6)
N3	0.0186 (7)	0.0256 (8)	0.0212 (7)	−0.0033 (6)	−0.0033 (6)	−0.0094 (6)
N4	0.0169 (6)	0.0182 (7)	0.0180 (7)	−0.0034 (5)	0.0000 (5)	−0.0067 (5)
C1	0.0204 (8)	0.0272 (9)	0.0201 (8)	−0.0070 (7)	−0.0018 (7)	−0.0053 (7)
C2	0.0279 (9)	0.0303 (10)	0.0222 (9)	−0.0042 (7)	0.0002 (7)	−0.0076 (8)
C3	0.0407 (11)	0.0258 (9)	0.0211 (9)	−0.0085 (8)	−0.0074 (8)	−0.0065 (7)
C4	0.0348 (10)	0.0340 (10)	0.0270 (9)	−0.0183 (8)	−0.0060 (8)	−0.0056 (8)
C5	0.0233 (9)	0.0352 (10)	0.0213 (9)	−0.0132 (7)	0.0009 (7)	−0.0059 (8)
C6	0.0199 (8)	0.0206 (8)	0.0163 (8)	−0.0041 (6)	−0.0031 (6)	−0.0036 (7)
C7	0.0177 (8)	0.0201 (8)	0.0207 (8)	−0.0041 (6)	0.0008 (6)	−0.0052 (7)
C8	0.0205 (8)	0.0204 (8)	0.0196 (8)	−0.0053 (6)	0.0023 (6)	−0.0084 (7)
C9	0.0179 (8)	0.0200 (8)	0.0185 (8)	−0.0054 (6)	0.0003 (6)	−0.0084 (7)
C10	0.0165 (7)	0.0203 (8)	0.0186 (8)	−0.0055 (6)	0.0018 (6)	−0.0071 (7)
C11	0.0212 (8)	0.0255 (9)	0.0292 (9)	−0.0010 (7)	−0.0036 (7)	−0.0160 (8)
C12	0.0244 (9)	0.0309 (10)	0.0253 (9)	−0.0064 (7)	−0.0042 (7)	−0.0141 (8)
C13	0.0181 (8)	0.0217 (8)	0.0162 (8)	−0.0061 (6)	0.0017 (6)	−0.0054 (6)
C14	0.0224 (8)	0.0186 (8)	0.0178 (8)	−0.0044 (6)	−0.0012 (7)	−0.0064 (7)
C15	0.0222 (9)	0.0257 (9)	0.0271 (9)	0.0008 (7)	−0.0066 (7)	−0.0099 (8)
C16	0.0251 (9)	0.0278 (10)	0.0356 (10)	0.0027 (7)	−0.0046 (8)	−0.0160 (8)
C17	0.0290 (9)	0.0237 (9)	0.0311 (10)	−0.0038 (7)	−0.0019 (8)	−0.0162 (8)
C18	0.0218 (8)	0.0205 (8)	0.0252 (9)	−0.0073 (7)	−0.0023 (7)	−0.0064 (7)
C19	0.0190 (8)	0.0201 (8)	0.0233 (8)	−0.0041 (6)	0.0005 (7)	−0.0081 (7)
C20	0.0673 (16)	0.0416 (12)	0.0410 (12)	−0.0169 (11)	−0.0073 (11)	−0.0226 (10)
C21	0.0207 (9)	0.0302 (10)	0.0326 (10)	0.0022 (7)	−0.0060 (8)	−0.0126 (8)
C22	0.0285 (9)	0.0221 (9)	0.0294 (9)	−0.0019 (7)	−0.0033 (8)	−0.0130 (7)
C23	0.0217 (9)	0.0282 (10)	0.0412 (11)	−0.0024 (7)	−0.0087 (8)	−0.0139 (8)
S1'	0.0201 (2)	0.0222 (2)	0.0229 (2)	−0.00219 (16)	−0.00535 (17)	−0.00906 (17)
O1'	0.0433 (8)	0.0365 (8)	0.0248 (7)	−0.0039 (6)	−0.0043 (6)	−0.0159 (6)
N1'	0.0207 (7)	0.0268 (8)	0.0279 (8)	−0.0031 (6)	−0.0071 (6)	−0.0064 (6)
N2'	0.0223 (7)	0.0244 (8)	0.0308 (8)	−0.0016 (6)	−0.0017 (6)	−0.0104 (7)
N3'	0.0215 (7)	0.0252 (8)	0.0238 (7)	−0.0068 (6)	−0.0044 (6)	−0.0081 (6)
N4'	0.0176 (7)	0.0187 (7)	0.0187 (7)	−0.0053 (5)	−0.0003 (5)	−0.0049 (5)
C1'	0.0254 (9)	0.0333 (10)	0.0225 (9)	−0.0136 (7)	−0.0051 (7)	−0.0040 (8)
C2'	0.0342 (10)	0.0330 (10)	0.0298 (10)	−0.0180 (8)	−0.0047 (8)	−0.0057 (8)
C3'	0.0375 (11)	0.0421 (12)	0.0317 (10)	−0.0235 (9)	0.0017 (8)	−0.0145 (9)
C4'	0.0332 (10)	0.0455 (12)	0.0233 (9)	−0.0225 (9)	−0.0010 (8)	−0.0057 (8)
C5'	0.0252 (9)	0.0315 (10)	0.0254 (9)	−0.0137 (7)	−0.0068 (7)	−0.0001 (8)
C6'	0.0160 (8)	0.0310 (10)	0.0280 (9)	−0.0091 (7)	−0.0041 (7)	−0.0071 (8)
C7'	0.0194 (8)	0.0235 (9)	0.0250 (9)	−0.0052 (7)	−0.0009 (7)	−0.0052 (7)
C8'	0.0240 (9)	0.0236 (9)	0.0286 (9)	−0.0041 (7)	0.0000 (7)	−0.0112 (7)
C9'	0.0229 (8)	0.0195 (8)	0.0200 (8)	−0.0057 (7)	−0.0022 (7)	−0.0062 (7)
C10'	0.0221 (8)	0.0208 (8)	0.0175 (8)	−0.0072 (6)	0.0006 (7)	−0.0050 (7)
C11'	0.0287 (9)	0.0236 (9)	0.0281 (9)	−0.0069 (7)	−0.0039 (8)	−0.0101 (7)
C12'	0.0310 (10)	0.0288 (10)	0.0245 (9)	−0.0114 (8)	−0.0038 (8)	−0.0114 (8)
C13'	0.0190 (8)	0.0224 (8)	0.0165 (8)	−0.0081 (6)	0.0017 (6)	−0.0043 (7)
C14'	0.0133 (7)	0.0231 (8)	0.0230 (8)	−0.0077 (6)	0.0025 (6)	−0.0070 (7)
C15'	0.0223 (9)	0.0244 (9)	0.0295 (9)	−0.0076 (7)	−0.0030 (7)	−0.0057 (7)
C16'	0.0289 (10)	0.0208 (9)	0.0463 (12)	−0.0051 (7)	−0.0039 (9)	−0.0105 (8)
C17'	0.0318 (10)	0.0302 (10)	0.0396 (11)	−0.0071 (8)	0.0013 (9)	−0.0213 (9)
C18'	0.0223 (8)	0.0304 (9)	0.0239 (9)	−0.0086 (7)	0.0015 (7)	−0.0114 (7)
C19'	0.0178 (8)	0.0220 (8)	0.0233 (8)	−0.0063 (6)	0.0033 (7)	−0.0082 (7)
C20'	0.091 (2)	0.0563 (15)	0.0408 (13)	−0.0408 (14)	0.0134 (13)	−0.0252 (11)
C21'	0.0256 (10)	0.0303 (10)	0.0405 (11)	0.0005 (8)	−0.0110 (9)	−0.0064 (9)
C22'	0.0319 (11)	0.0401 (12)	0.0520 (13)	0.0012 (9)	−0.0059 (10)	−0.0285 (10)
C23'	0.0325 (10)	0.0335 (10)	0.0235 (9)	−0.0081 (8)	−0.0051 (8)	−0.0073 (8)
N5	0.0193 (7)	0.0212 (7)	0.0225 (7)	−0.0002 (6)	−0.0070 (6)	−0.0083 (6)
N5'	0.0179 (7)	0.0220 (7)	0.0209 (7)	−0.0035 (5)	−0.0043 (6)	−0.0064 (6)

### Geometric parameters (Å, °)

**Table:**

S1—C7	1.7377 (16)	S1'—C7'	1.7427 (17)
S1—C9	1.7414 (16)	O1'—C18'	1.372 (2)
O1—C18	1.3739 (19)	O1'—C23'	1.416 (2)
O1—C23	1.4259 (19)	N1'—C7'	1.366 (2)
N1—C7	1.362 (2)	N1'—C6'	1.416 (2)
N1—C6	1.418 (2)	N1'—C21'	1.474 (2)
N1—C21	1.4709 (19)	N2'—C7'	1.309 (2)
N2—C7	1.314 (2)	N2'—C8'	1.371 (2)
N2—C8	1.365 (2)	N3'—C12'	1.336 (2)
N3—C12	1.330 (2)	N3'—C13'	1.356 (2)
N3—C13	1.352 (2)	N4'—C13'	1.336 (2)
N4—C13	1.3362 (19)	N4'—C10'	1.356 (2)
N4—C10	1.3563 (19)	C1'—C2'	1.385 (2)
C1—C2	1.380 (2)	C1'—C6'	1.388 (2)
C1—C6	1.391 (2)	C1'—H1'	0.9500
C1—H1	0.9500	C2'—C3'	1.389 (3)
C2—C3	1.387 (3)	C2'—H2'	0.9500
C2—H2	0.9500	C3'—C4'	1.385 (3)
C3—C4	1.391 (3)	C3'—C20'	1.499 (3)
C3—C20	1.513 (2)	C4'—C5'	1.377 (3)
C4—C5	1.382 (2)	C4'—H4'	0.9500
C4—H4	0.9500	C5'—C6'	1.395 (2)
C5—C6	1.393 (2)	C5'—H5'	0.9500
C5—H5	0.9500	C8'—C9'	1.374 (2)
C8—C9	1.381 (2)	C8'—C22'	1.502 (2)
C8—C22	1.503 (2)	C9'—C10'	1.450 (2)
C9—C10	1.448 (2)	C10'—C11'	1.393 (2)
C10—C11	1.396 (2)	C11'—C12'	1.374 (2)
C11—C12	1.372 (2)	C11'—H11'	0.9500
C11—H11	0.9500	C12'—H12'	0.9500
C12—H12	0.9500	C13'—N5'	1.369 (2)
C13—N5	1.370 (2)	C14'—C15'	1.390 (2)
C14—C19	1.395 (2)	C14'—C19'	1.396 (2)
C14—N5	1.398 (2)	C14'—N5'	1.402 (2)
C14—C15	1.404 (2)	C15'—C16'	1.380 (2)
C15—C16	1.379 (2)	C15'—H15'	0.9500
C15—H15	0.9500	C16'—C17'	1.376 (3)
C16—C17	1.381 (2)	C16'—H16'	0.9500
C16—H16	0.9500	C17'—C18'	1.383 (2)
C17—C18	1.387 (2)	C17'—H17'	0.9500
C17—H17	0.9500	C18'—C19'	1.386 (2)
C18—C19	1.388 (2)	C19'—H19'	0.9500
C19—H19	0.9500	C20'—H20D	0.9800
C20—H20A	0.9800	C20'—H20E	0.9800
C20—H20B	0.9800	C20'—H20F	0.9800
C20—H20C	0.9800	C21'—H21D	0.9800
C21—H21A	0.9800	C21'—H21E	0.9800
C21—H21B	0.9800	C21'—H21F	0.9800
C21—H21C	0.9800	C22'—H22D	0.9800
C22—H22A	0.9800	C22'—H22E	0.9800
C22—H22B	0.9800	C22'—H22F	0.9800
C22—H22C	0.9800	C23'—H23D	0.9800
C23—H23A	0.9800	C23'—H23E	0.9800
C23—H23B	0.9800	C23'—H23F	0.9800
C23—H23C	0.9800	N5—H0	0.8800
S1'—C9'	1.7367 (16)	N5'—H0'	0.8800
			
C7—S1—C9	88.64 (8)	C7'—N1'—C21'	115.57 (15)
C18—O1—C23	116.99 (13)	C6'—N1'—C21'	119.97 (14)
C7—N1—C6	124.09 (13)	C7'—N2'—C8'	110.83 (14)
C7—N1—C21	116.77 (14)	C12'—N3'—C13'	114.45 (14)
C6—N1—C21	119.14 (13)	C13'—N4'—C10'	116.72 (14)
C7—N2—C8	110.62 (13)	C2'—C1'—C6'	120.40 (16)
C12—N3—C13	114.57 (13)	C2'—C1'—H1'	119.8
C13—N4—C10	117.02 (14)	C6'—C1'—H1'	119.8
C2—C1—C6	119.79 (17)	C1'—C2'—C3'	121.91 (18)
C2—C1—H1	120.1	C1'—C2'—H2'	119.0
C6—C1—H1	120.1	C3'—C2'—H2'	119.0
C1—C2—C3	122.08 (17)	C4'—C3'—C2'	116.98 (18)
C1—C2—H2	119.0	C4'—C3'—C20'	121.47 (18)
C3—C2—H2	119.0	C2'—C3'—C20'	121.55 (18)
C2—C3—C4	117.55 (17)	C5'—C4'—C3'	122.02 (17)
C2—C3—C20	121.09 (19)	C5'—C4'—H4'	119.0
C4—C3—C20	121.34 (19)	C3'—C4'—H4'	119.0
C5—C4—C3	121.29 (17)	C4'—C5'—C6'	120.58 (17)
C5—C4—H4	119.4	C4'—C5'—H5'	119.7
C3—C4—H4	119.4	C6'—C5'—H5'	119.7
C4—C5—C6	120.32 (17)	C1'—C6'—C5'	118.04 (16)
C4—C5—H5	119.8	C1'—C6'—N1'	121.29 (15)
C6—C5—H5	119.8	C5'—C6'—N1'	120.66 (16)
C1—C6—C5	118.96 (16)	N2'—C7'—N1'	121.33 (15)
C1—C6—N1	121.09 (15)	N2'—C7'—S1'	115.06 (12)
C5—C6—N1	119.88 (15)	N1'—C7'—S1'	123.46 (13)
N2—C7—N1	121.04 (14)	N2'—C8'—C9'	115.64 (15)
N2—C7—S1	115.41 (12)	N2'—C8'—C22'	116.69 (15)
N1—C7—S1	123.51 (13)	C9'—C8'—C22'	127.65 (16)
N2—C8—C9	115.91 (14)	C8'—C9'—C10'	131.90 (15)
N2—C8—C22	115.90 (14)	C8'—C9'—S1'	109.75 (12)
C9—C8—C22	128.19 (15)	C10'—C9'—S1'	118.34 (12)
C8—C9—C10	131.61 (15)	N4'—C10'—C11'	120.66 (14)
C8—C9—S1	109.42 (12)	N4'—C10'—C9'	115.53 (14)
C10—C9—S1	118.95 (12)	C11'—C10'—C9'	123.78 (15)
N4—C10—C11	120.22 (14)	C12'—C11'—C10'	117.26 (16)
N4—C10—C9	116.07 (14)	C12'—C11'—H11'	121.4
C11—C10—C9	123.70 (14)	C10'—C11'—H11'	121.4
C12—C11—C10	117.24 (15)	N3'—C12'—C11'	123.97 (16)
C12—C11—H11	121.4	N3'—C12'—H12'	118.0
C10—C11—H11	121.4	C11'—C12'—H12'	118.0
N3—C12—C11	124.25 (15)	N4'—C13'—N3'	126.86 (15)
N3—C12—H12	117.9	N4'—C13'—N5'	119.53 (14)
C11—C12—H12	117.9	N3'—C13'—N5'	113.61 (14)
N4—C13—N3	126.68 (14)	C15'—C14'—C19'	119.57 (15)
N4—C13—N5	120.21 (14)	C15'—C14'—N5'	116.54 (14)
N3—C13—N5	113.11 (13)	C19'—C14'—N5'	123.74 (14)
C19—C14—N5	124.14 (14)	C16'—C15'—C14'	120.21 (16)
C19—C14—C15	119.27 (15)	C16'—C15'—H15'	119.9
N5—C14—C15	116.57 (14)	C14'—C15'—H15'	119.9
C16—C15—C14	120.50 (16)	C17'—C16'—C15'	120.70 (17)
C16—C15—H15	119.7	C17'—C16'—H16'	119.6
C14—C15—H15	119.7	C15'—C16'—H16'	119.6
C15—C16—C17	120.48 (16)	C16'—C17'—C18'	119.15 (17)
C15—C16—H16	119.8	C16'—C17'—H17'	120.4
C17—C16—H16	119.8	C18'—C17'—H17'	120.4
C16—C17—C18	119.04 (16)	O1'—C18'—C17'	115.16 (15)
C16—C17—H17	120.5	O1'—C18'—C19'	123.49 (15)
C18—C17—H17	120.5	C17'—C18'—C19'	121.34 (16)
O1—C18—C17	115.16 (15)	C18'—C19'—C14'	118.95 (15)
O1—C18—C19	123.15 (14)	C18'—C19'—H19'	120.5
C17—C18—C19	121.68 (15)	C14'—C19'—H19'	120.5
C18—C19—C14	118.98 (15)	C3'—C20'—H20D	109.5
C18—C19—H19	120.5	C3'—C20'—H20E	109.5
C14—C19—H19	120.5	H20D—C20'—H20E	109.5
C3—C20—H20A	109.5	C3'—C20'—H20F	109.5
C3—C20—H20B	109.5	H20D—C20'—H20F	109.5
H20A—C20—H20B	109.5	H20E—C20'—H20F	109.5
C3—C20—H20C	109.5	N1'—C21'—H21D	109.5
H20A—C20—H20C	109.5	N1'—C21'—H21E	109.5
H20B—C20—H20C	109.5	H21D—C21'—H21E	109.5
N1—C21—H21A	109.5	N1'—C21'—H21F	109.5
N1—C21—H21B	109.5	H21D—C21'—H21F	109.5
H21A—C21—H21B	109.5	H21E—C21'—H21F	109.5
N1—C21—H21C	109.5	C8'—C22'—H22D	109.5
H21A—C21—H21C	109.5	C8'—C22'—H22E	109.5
H21B—C21—H21C	109.5	H22D—C22'—H22E	109.5
C8—C22—H22A	109.5	C8'—C22'—H22F	109.5
C8—C22—H22B	109.5	H22D—C22'—H22F	109.5
H22A—C22—H22B	109.5	H22E—C22'—H22F	109.5
C8—C22—H22C	109.5	O1'—C23'—H23D	109.5
H22A—C22—H22C	109.5	O1'—C23'—H23E	109.5
H22B—C22—H22C	109.5	H23D—C23'—H23E	109.5
O1—C23—H23A	109.5	O1'—C23'—H23F	109.5
O1—C23—H23B	109.5	H23D—C23'—H23F	109.5
H23A—C23—H23B	109.5	H23E—C23'—H23F	109.5
O1—C23—H23C	109.5	C13—N5—C14	131.15 (13)
H23A—C23—H23C	109.5	C13—N5—H0	114.4
H23B—C23—H23C	109.5	C14—N5—H0	114.4
C9'—S1'—C7'	88.66 (8)	C13'—N5'—C14'	129.69 (13)
C18'—O1'—C23'	118.32 (13)	C13'—N5'—H0'	115.2
C7'—N1'—C6'	122.53 (14)	C14'—N5'—H0'	115.2
			
C6—C1—C2—C3	−0.4 (3)	C20'—C3'—C4'—C5'	178.9 (2)
C1—C2—C3—C4	−0.5 (3)	C3'—C4'—C5'—C6'	0.1 (3)
C1—C2—C3—C20	−178.81 (17)	C2'—C1'—C6'—C5'	−3.3 (3)
C2—C3—C4—C5	1.0 (3)	C2'—C1'—C6'—N1'	176.88 (16)
C20—C3—C4—C5	179.31 (17)	C4'—C5'—C6'—C1'	2.2 (3)
C3—C4—C5—C6	−0.6 (3)	C4'—C5'—C6'—N1'	−178.01 (16)
C2—C1—C6—C5	0.8 (2)	C7'—N1'—C6'—C1'	−46.7 (3)
C2—C1—C6—N1	177.79 (14)	C21'—N1'—C6'—C1'	149.80 (17)
C4—C5—C6—C1	−0.3 (2)	C7'—N1'—C6'—C5'	133.47 (18)
C4—C5—C6—N1	−177.33 (15)	C21'—N1'—C6'—C5'	−30.0 (2)
C7—N1—C6—C1	48.2 (2)	C8'—N2'—C7'—N1'	174.26 (16)
C21—N1—C6—C1	−131.79 (17)	C8'—N2'—C7'—S1'	−1.4 (2)
C7—N1—C6—C5	−134.82 (18)	C6'—N1'—C7'—N2'	−177.96 (16)
C21—N1—C6—C5	45.2 (2)	C21'—N1'—C7'—N2'	−13.8 (3)
C8—N2—C7—N1	−178.21 (15)	C6'—N1'—C7'—S1'	−2.7 (3)
C8—N2—C7—S1	−0.28 (19)	C21'—N1'—C7'—S1'	161.47 (14)
C6—N1—C7—N2	−172.95 (15)	C9'—S1'—C7'—N2'	2.15 (15)
C21—N1—C7—N2	7.1 (2)	C9'—S1'—C7'—N1'	−173.40 (17)
C6—N1—C7—S1	9.3 (2)	C7'—N2'—C8'—C9'	−0.5 (2)
C21—N1—C7—S1	−170.69 (13)	C7'—N2'—C8'—C22'	177.95 (17)
C9—S1—C7—N2	0.00 (14)	N2'—C8'—C9'—C10'	−178.95 (18)
C9—S1—C7—N1	177.87 (16)	C22'—C8'—C9'—C10'	2.9 (3)
C7—N2—C8—C9	0.5 (2)	N2'—C8'—C9'—S1'	2.0 (2)
C7—N2—C8—C22	−178.81 (15)	C22'—C8'—C9'—S1'	−176.16 (17)
N2—C8—C9—C10	−178.73 (17)	C7'—S1'—C9'—C8'	−2.23 (14)
C22—C8—C9—C10	0.5 (3)	C7'—S1'—C9'—C10'	178.60 (15)
N2—C8—C9—S1	−0.52 (19)	C13'—N4'—C10'—C11'	−2.3 (2)
C22—C8—C9—S1	178.71 (15)	C13'—N4'—C10'—C9'	179.90 (15)
C7—S1—C9—C8	0.28 (13)	C8'—C9'—C10'—N4'	−167.75 (18)
C7—S1—C9—C10	178.75 (14)	S1'—C9'—C10'—N4'	11.2 (2)
C13—N4—C10—C11	0.7 (2)	C8'—C9'—C10'—C11'	14.5 (3)
C13—N4—C10—C9	−179.93 (15)	S1'—C9'—C10'—C11'	−166.56 (14)
C8—C9—C10—N4	177.00 (17)	N4'—C10'—C11'—C12'	2.5 (3)
S1—C9—C10—N4	−1.1 (2)	C9'—C10'—C11'—C12'	−179.83 (17)
C8—C9—C10—C11	−3.7 (3)	C13'—N3'—C12'—C11'	−1.8 (3)
S1—C9—C10—C11	178.22 (14)	C10'—C11'—C12'—N3'	−0.4 (3)
N4—C10—C11—C12	−1.0 (3)	C10'—N4'—C13'—N3'	−0.2 (3)
C9—C10—C11—C12	179.69 (17)	C10'—N4'—C13'—N5'	179.63 (15)
C13—N3—C12—C11	1.3 (3)	C12'—N3'—C13'—N4'	2.2 (3)
C10—C11—C12—N3	−0.1 (3)	C12'—N3'—C13'—N5'	−177.63 (15)
C10—N4—C13—N3	0.7 (3)	C19'—C14'—C15'—C16'	−2.9 (3)
C10—N4—C13—N5	−179.70 (15)	N5'—C14'—C15'—C16'	172.79 (16)
C12—N3—C13—N4	−1.7 (3)	C14'—C15'—C16'—C17'	1.1 (3)
C12—N3—C13—N5	178.67 (15)	C15'—C16'—C17'—C18'	1.3 (3)
C19—C14—C15—C16	0.9 (3)	C23'—O1'—C18'—C17'	−173.22 (17)
N5—C14—C15—C16	−178.00 (16)	C23'—O1'—C18'—C19'	7.4 (3)
C14—C15—C16—C17	−1.7 (3)	C16'—C17'—C18'—O1'	178.81 (18)
C15—C16—C17—C18	0.5 (3)	C16'—C17'—C18'—C19'	−1.8 (3)
C23—O1—C18—C17	158.88 (17)	O1'—C18'—C19'—C14'	179.33 (16)
C23—O1—C18—C19	−22.2 (2)	C17'—C18'—C19'—C14'	0.0 (3)
C16—C17—C18—O1	−179.50 (16)	C15'—C14'—C19'—C18'	2.3 (2)
C16—C17—C18—C19	1.5 (3)	N5'—C14'—C19'—C18'	−173.03 (15)
O1—C18—C19—C14	178.81 (15)	N4—C13—N5—C14	−2.6 (3)
C17—C18—C19—C14	−2.3 (3)	N3—C13—N5—C14	177.09 (16)
N5—C14—C19—C18	179.88 (15)	C19—C14—N5—C13	20.4 (3)
C15—C14—C19—C18	1.1 (3)	C15—C14—N5—C13	−160.71 (17)
C6'—C1'—C2'—C3'	2.3 (3)	N4'—C13'—N5'—C14'	−12.8 (3)
C1'—C2'—C3'—C4'	0.0 (3)	N3'—C13'—N5'—C14'	167.01 (16)
C1'—C2'—C3'—C20'	179.9 (2)	C15'—C14'—N5'—C13'	164.55 (17)
C2'—C3'—C4'—C5'	−1.2 (3)	C19'—C14'—N5'—C13'	−20.0 (3)

### Hydrogen-bond geometry (Å, °)

**Table:**

*D*—H···*A*	*D*—H	H···*A*	*D*···*A*	*D*—H···*A*
N5—H0···N3'^i^	0.88	2.27	3.151 (2)	177
N5'—H0'···N3^i^	0.88	2.24	3.089 (2)	164
C22—H22B···O1^ii^	0.98	2.57	3.500 (2)	159

Symmetry codes: (i) −*x*, −*y*+1, −*z*; (ii) −*x*+1, −*y*+1, −*z*.
